# Vitamin D and hypophosphatemia in patients with anorexia nervosa and avoidant/restrictive food intake disorder: a case control study

**DOI:** 10.1186/s40337-023-00913-w

**Published:** 2023-11-02

**Authors:** Meredith R. Kells, Chloe Roske, Ashlie Watters, Leah Puckett, Jennifer E. Wildes, Scott J. Crow, Philip S. Mehler

**Affiliations:** 1 Department of Psychiatry and Behavioral Neuroscience, University of Chicago, Chicago, IL USA; 2 School of Nursing, University of Rochester, Rochester, NY USA; 3 Department of Psychiatry and Behavioral Sciences, Albert Einstein College of Medicine, Bronx, NY USA; 4 ACUTE Center for Eating Disorders and Severe Malnutrition, Denver, CO USA; 5 University of Colorado School of Medicine, Aurora, CO USA; 6 University of Minnesota, Minneapolis, MN USA; 7 The Emily Program, St. Paul, MN USA

**Keywords:** Eating disorders, Refeeding syndrome, Hypophosphatemia, 25-hydroxy vitamin D, Inpatient, Malnutrition

## Abstract

**Background:**

Refeeding hypophosphatemia (RH) is a common complication of nutritional restoration in malnourished individuals, yet clear risk stratification remains elusive. Individuals with anorexia nervosa (AN) and avoidant/restrictive food intake disorder (ARFID) may be deficient in vitamin D, an important component of dietary phosphorus absorption in the gut. The relationship between vitamin D and RH in AN and ARFID is unknown. Therefore, the aims of this study were to (1) report rates of low serum 25-hydroxy vitamin D and RH in AN and ARFID; (2) describe associations between phosphorus and variables associated with RH identified in extant literature; (3) examine the relationship between 25-hydroxy vitamin D and RH and (4) investigate moderation by vitamin D between variables of interest and phosphorus level.

**Method:**

Analyses included retrospective chart review of 307 individuals admitted to the ACUTE Center for Eating Disorders and Severe Malnutrition with a diagnosis of AN or ARFID. Variables of interest included admission laboratory values (vitamin D level, comprehensive metabolic panel, hemoglobin, point-of-care blood glucose), anthropometric measures (weight, body mass index [BMI], % ideal body weight [IBW]), age, duration of illness, length of stay, feeding method, and serum phosphorus nadir. Pearson and Spearman rank correlation, one-way ANOVA, and regression analyses were used to determine the relationship between variables and serum phosphorus.

**Results:**

Over 1/3 of the sample (35.3%) had serum phosphorus levels ≤ 2.9 mg/dL. There were no significant differences between groups in phosphorus nadir (*p* = .17, *η*^2^ = 0.12) or hypophosphatemia (*p* = .16, ϕc = 0.11). Thirty-five (35%) of individuals with ARFID were either deficient or insufficient in vitamin D, compared to 29% of individuals with AN. Individuals with AN had significantly higher mean vitamin D levels compared to those with ARFID (*p* = .03; *η*^2^ = 0.015). Nadir phosphorus showed a positive association with weight, BMI, %IBW, potassium, and calcium on admission, and a negative association with length of stay, hemoglobin, and total number of tube-fed days. Higher levels of 25-hydroxy vitamin D moderated the relationship between serum phosphorus nadir and weight on admission (*p* = .0004).

**Conclusion:**

Individuals diagnosed with ARFID are as nutritionally fragile as those with AN regarding vitamin D and RH. The negative feedback loop involving vitamin D that maintains phosphorus homeostasis may play a role in the development of RH in AN and ARFID.

## Abstract (plain language summary)

Refeeding hypophosphatemia (RH) is a common and potentially serious complication of nutrition restoration, yet its risk is not fully understood. Vitamin D is an important part of phosphorus absorption in the gut. We examined 25-hydroxy vitamin D levels on admission and the relationship with RH in individuals with avoidant/restrictive food intake disorder (ARFID) and anorexia nervosa (AN). Results showed individuals with ARFID had significantly lower vitamin D levels than individuals with restrictive type AN, but not individuals with binge/purge type AN. Additionally, analyses showed that higher levels of vitamin D may play a role in the association between RH and weight on admission. Better understanding of RH risk may improve care.

## Introduction

Individuals with anorexia nervosa (AN) and avoidant/restrictive food intake disorder (ARFID) may require medical hospitalization for safe restoration of nutrition due to weight loss, growth failure, or medical instability [1–6]. During hospitalization, one critical area of clinical concern is refeeding syndrome, a potentially life-threatening cluster of metabolic and electrolyte abnormalities that can occur when a person in a starvation state shifts from catabolism to anabolism during nutrition restoration [7]. Refeeding syndrome is associated with impaired respiratory and cardiac function, hemolysis, rhabdomyolysis, encephalopathy, fluid overload, and death [7]. Concerningly, studies have identified that protocols aimed at reducing risk through conservative nutrition restoration may be of little utility and lead to poorer weight gain [8–10]. Therefore, understanding who is most at risk is crucial to providing proper treatment to patients with AN and ARFID [11–13].

One key etiological factor for the development of refeeding syndrome, and a main target of clinical monitoring, is low serum phosphorus level, known as refeeding hypophosphatemia (RH) [14]. RH has been observed in up to 38% of individuals with AN [15, 51]. Although many factors have suggested associations with RH (e.g. low body mass index [BMI], rate and magnitude of weight loss, low serum magnesium and potassium, higher levels of hemoglobin), [15–23] they are not sufficient to understand and identify who is at greatest risk for RH in populations of AN. Additionally, there has been little investigation into the development of RH in individuals with ARFID. One area yet to be fully investigated is the relationship between vitamin D and RH in this population.

People with AN and ARFID may be deficient in vitamin D, a fat-soluble vitamin obtained from sun exposure and nutritional sources [24–26]. The Endocrine Society recommends serum 25-hydroxy vitamin D as a reliable measure of vitamin D, with levels below 20 ng/mL classified as deficient, levels between 21 and 29 classified as insufficient, and levels 30 or greater classified as sufficient [27]. Studies have reported mixed findings related to vitamin D levels in individuals with AN, with some showing high prevalence of vitamin D deficiency or insufficiency in hospitalized individuals and others showing higher levels of vitamin D compared to controls [24, 28–32]. Low 25-hydroxy vitamin D levels have also been described in those with ARFID [26, 33]. 

The role of vitamin D in osteoporosis and osteopenia has been a topic of interest in literature pertaining to eating disorders, but has yet to be described in this population with regards to RH [26, 34, 35]. As a component of a negative feedback loop involving phosphorus, calcium, parathyroid hormone, and fibroblast growth factor 23, vitamin D activates sodium-phosphate co-transporters on intestinal epithelial cells that are responsible for phosphorus absorption in the gut [36, 37]. Therefore, low levels of 25-hydroxy vitamin D may play a role in the development of RH and risk of refeeding syndrome.

## Current study

Due to vitamin D’s potential role in the development of RH, the aims of this study were:

Aim 1: Report rates of low serum 25-hydroxy vitamin D levels and refeeding hypophosphatemia in AN and ARFID.

Hypothesis: Individuals with AN will have lower phosphorus nadir, lower mean vitamin D on admission, and greater likelihood to experience RH and vitamin D deficiency than individuals with ARFID.

Aim 2: Describe associations between nadir phosphorus level and variables associated with hypophosphatemia in the literature (admission labs, weight on admission, % IBW on admission, length of stay, nasogastric tube feeding, age, caloric prescription, and duration of illness).

 Hypothesis: Nadir phosphorus level will show positive correlations with weight on admission, %IBW, and admission potassium and magnesium and negative correlations with length of stay, nasogastric tube feeding, age, caloric prescription, admission AST and ALT, and duration of illness.

Aim 3: Examine the relationship between admission serum 25-hydroxy vitamin D level and refeeding hypophosphatemia during nutritional rehabilitation for individuals with AN and ARFID

Hypothesis: Lower level of admission 25-hydroxy vitamin D will be associated with refeeding hypophosphatemia during nutritional rehabilitation.

Exploratory Aim: As previous studies have described a low variance explained by variables associated with hypophosphatemia, we also sought to investigate whether 25-hydroxy vitamin D level moderated the relationship between variables and serum nadir phosphorus level.

## Methods

Analyses included 307 medical charts representing the initial presentation of individuals admitted to the ACUTE Center for Eating Disorders and Severe Malnutrition (ACUTE) at Denver Health Hospital with a diagnosis of anorexia nervosa restricting subtype (AN-R), anorexia nervosa binge-eating/purging subtype (AN-BP) and avoidant/restrictive food intake disorder (ARFID) between June 1, 2016 and July 31, 2021. ACUTE is a thirty-bed critical care unit for individuals with serious medical complications resulting from extreme forms of disordered eating. Patients admitted to ACUTE are at least 15 years of age and meet any or all of the following criteria: < 70% of the patient’s ideal body weight (IBW), BMI less than 15 kg/m [2], a history of severe medical complications of an eating disorder (e.g., fluid and/or electrolyte derangements, refeeding syndrome, organ failure, cardiac abnormalities or unstable vital signs), or required monitored cessation of purging behaviors. Previous research in the population at ACUTE revealed a RH prevalence of 33.3% [16]. Exclusion criteria for the current study included other eating disorder diagnoses (e.g., binge-eating disorder, bulimia nervosa), less than 18 years of age, parenteral nutrition administration at any point during hospitalization, diagnosis of malabsorptive disease (e.g., celiac, short gut), chronic liver or renal disease, history of bariatric surgery, length of stay of less than 3 days, and transfer from an outside hospital. Existing medical records were examined for variables of interest including serum phosphorus nadir, serum 25-hydroxy vitamin D level on admission, weight and BMI, % IBW, duration of illness, length of stay, nasogastric tube feeding, caloric prescription on admission, age, admission comprehensive metabolic panel, admission point-of-care blood glucose level, and serum hemoglobin. Percent IBW was calculated using the Hamwi method [53]. Laboratory analyses for serum phosphorus, 25-hydroxy vitamin D, comprehensive metabolic panel, and serum hemoglobin were performed at the Denver Heath laboratory as part of standard procedures, including all routine and existing quality assurance analyses. Point-of-care blood glucose level was performed at the bedside using glucometer operated by trained and certified unit staff. Biological sex was not included in the analyses due to an insufficient number of males in this cohort.

Patients at ACUTE were started on oral nutrition restoration unless oral feeding was not possible. Nasogastric tube feeding was used for oral food refusal on a short-term basis and discontinued as soon as an individual could tolerate oral intake. Electrolytes were monitored daily and replenished via oral supplementation at the discretion and clinical judgement of the provider. Intravenous electrolyte repletion was necessary in cases where rapid or dangerous drop in levels was detected.

### Statistical analyses

Descriptive statistics were used to characterize the sample. As serum phosphorus nadir may influence clinical decision making even without meeting the threshold for hypophosphatemia (e.g. increase or start supplemental phosphorus if serum phosphorus is down trending), we examined both serum phosphorus nadir and the presence or absence hypophosphatemia for each study aim. To address Aim 1, descriptive statistics and analysis of variance (ANOVA) were used. Tukey post-hoc analyses were performed for significant relationships, as appropriate, and reported in Table 2. To address Aim 2, Pearson’s correlations and Spearman rank correlations were used to examine associations between nadir phosphorus and variables of interest. To address Aim 3, binary logistic regression was used to test the hypothesis that individuals with lower 25-hydroxy vitamin D level on admission would be more likely to demonstrate refeeding hypophosphatemia (defined as serum phosphorus < 2.9 mg/dL) during hospitalization. Additionally, a logistic regression model was created with variables significantly associated with the outcome of hypophosphatemia (Aim 2) and vitamin D, using a *p*-value of < 0.10; all variables were entered into the model simultaneously (Table 4). To address the exploratory aim, moderation analyses were run to examine interactions between variables of interest and vitamin D on the outcome of nadir phosphorus using PROCESS macro package [38]. Effect sizes were reported as partial eta squared (*η*^2^, small = 0.01, medium = 0.06, large = 0.14), and Cramer’s V (ϕc; small = 0.10, medium = 0.30, large = 0.50) [52]. Statistical analyses were conducted using SPSS version 27 statistical software [39], and *p*-values of < 0.05 were considered statistically significant. Two individuals were missing vitamin D levels and were not included in analyses (one ARFID, one AN-R). This study was approved by the Colorado Multiple Institutional Review Board.

## Results

The mean age of the study cohort was 29 years (SD = 11.42), and the sample was overwhelmingly female (Table 1). There were no significant differences between individuals with AN and individuals with ARFID in age, length of stay, admit weight, admit BMI, admit %IBW, duration of illness, or caloric prescription.

**Table 1:** Patient characteristics

	Whole sample (*n* = 307)		ARFID (*n* = 58)		AN (*n* = 249)	
	Mean (SD)	Range	Mean (SD)	Range	Mean (SD)	Range
Age (years)	29.8 (11.42)	18–63	28.9 (12.7)	18–63	30.0 (11)	18–63
Length of stay (days)	18.6 (9.72)	3–80	19.7 (9.5)	3–44	18.4 (9.8)	3–80
Admit weight (Kg)	40.9 (7.63)	24.9–70.9	41.2 (8.2)	23–70.5	40.8 (7.5)	24.9–70.9
Admit BMI (kg/m2)	14.7 (2.16)	9.67–26.42	14.8 (2.0)	10.7–20.9	14.7 (2.1)	9.7–26.4
Admit IBW (%)	70.2 (10.32)	45.73–127.60	70.2 (9.8)	50–103	70.2 (10.4)	45.7–127.6
Weight Gain (kg/week)	1.63 (.84)	−2.2–5.0	1.3 (.77)	−2.2–3.2	1.7 (.84)	−1.87–5.0
Duration of illness (years)	12.5 (10.21)	1–48	5.8 (5.9)	1–23	13.6 (10.3)	1–48
Admit calories prescribed (kcal)	1462	1400–3400	1515 (373)	1400–4000	1449 (196)	1400–3400
Admit Magnesium	1.8 (.22)	1.1–2.4	1.8 (.22)	1.2–2.3	1.8 (.22)	1.1–2.4
Admit Calcium	8.9 (.53)	7.0–10.8	8.9 (.50)	8.0–10.4	8.9 (.54)	7.0–10.8
Admit Potassium	3.7 (.52)	1.6–5.0	3.7 (.47)	2.2–4.4	3.7 (.54)	1.6–5.0
Admit POCT Blood Glucose	69.7 (18.3)	31–244	77.3 (16.2)	37–244	69.7 (15.4)	31–174
AST	24.1 (13.0)	7–133	23.6 (13.7)	8–81	24.1 (12.9)	7–133
ALT	26.3 (17.5)	7–212	30.1 (29.9)	8–212	25.5 (13.1)	7–11
Admit Hemoglobin	12.9 (1.7)	6.4–17.0	12.9 (1.7)	8.1–16.4	12.9 (1.7)	6.4–17.0
Admit Prealbumin	20.9 (6.3)	4.8–48.2	20.4 (6.3)	8.0–38.6	21.0 (6.3)	4.8–48.2
Length of Stay	18.6 (9.7)	3–80	19.7 (9.5)	3–44	18.4 (.84)	3–80
# of Tube Fed Days	3.7 (7.9)	0–39	4.7 (8.3)	0–38	3.5 (7.8)	0–39

	Whole sample (*n* = 307)		ARFID (*n* = 58)		AN (*n* = 249)	
	*n* %		*n* %		*n* %	
Female	280	91.2	47	81	233	93.6
Male	27	8.8	11	19	16	6.4

*ARFID* Avoidant/restrictive food intake disorder, *AN* Anorexia nervosa, *BMI* Body mass index, *IBW* Ideal body weight

### Nadir serum phosphorus and refeeding hypophosphatemia by diagnosis

Over one-third of the patients (*n* = 109, 35.3%) recorded a serum phosphorus of 2.9 mg/dL or less over the course of admission. Hypophosphatemia during admission was found in 44% of individuals with ARFID compared to 33% individuals with AN (Table 2). There were no significant differences between groups with respect to mean phosphorus nadir (*F*[2,304] = 1.80, *p* = 0.17, *η*^2^ = 0.12) or prevalence of hypophosphatemia (*x*^*2*^[1] = 3.67, *p* = 0.16, ϕc = 0.11).

**Table 2:** Phosphorus and vitamin D on admission by ARFID, AN with 3-way post-hoc analyses

	Whole sample (*N* = 307)	ARFID (*n* = 57)	AN-Both Subtype (*n* = 248)	AN-R(*n* = 112)	AN-BP(*n* = 136)				
**Phosphorus**						*F*	*p*	*η*^*2*^	
Nadir Mean (SD) mg/dL	3.12 (.3)	3.06 (.64)	3.14 (.63)	3.21 (.53)	3.1 (.70)	1.809	.166	0.12	
Nadir Range mg/dL	.5–5.2	1.4–4.2	.5–5.2	2.2–4.9	.5–5.2				
						*x*^*2*^	*p*	ϕc	
Hypophosphatemia n (%)	109 (35.5)	26 (44.8)	83 (33.3)	31 (30.1%)	49 (36)	3.66	.161	0.11	
**Vitamin D**						*F*	*p*	*η*^*2*^	*Post-hoc Tukey test*
Mean (SD) (ng/mL)	39.1 (17.6)	36.1 (14.1)	41.6 (18.1)	44.1 (18.0)	39.6 (17.9)	6.79	.001	0.044	AN-R > AN-BP (*p* = .009)
Range (ng/mL)	9–99	10–85	9–99	13–89	9–99				AN-R > ARFID (*p* = .004)
Deficient* *n* (%)	22 (7)	5 (9)	17 (7)	7 (6.3)	10 (7.4)				AN-BP > ARFID (*p* = .642)
Insufficient^#^ *n* (%)	69 (23)	15 (26)	54 (22)	22 (19.6)	32 (23.5)				
Sufficient^^^ *n* (%)	214 (70)	37 (65)	172 (71)	83 (74.1)	94 (69.1)				

*ARFID* Avoidant/restrictive food intake disorder, *AN* Anorexia nervosa; *AN-R* Anorexia nervosa, restricting subtype; *AN-BP* Anorexia nervosa, binge/purge subtype

Vitamin D level definition as follows [27]:

^*^Deficient =  ≤ 20 ng/mL

^#^Insufficient = 21–29 ng/mL

^^^Sufficient =  ≥ 30 ng/mL

### Vitamin D status by diagnosis

Mean (SD) admission 25-hydroxy vitamin D level was 40.6 ng/mL (17.6) in the sample overall. Admission 25-hydroxy vitamin D levels differed significantly between diagnoses (F[2,294] = 6.79, *p* = 0.001; *η*^2^ = 0.044); post-hoc analyses showed significantly higher 25-hydroxy vitamin D levels in those diagnosed with AN-R (mean 44.1 ng/mL) compared to individuals with AN-BP (mean = 39.6 ng/mL; *p* = 0.009), as well as significantly higher levels in those with AN-R compared to ARFID (mean = 36.1 ng/mL; *p* = 0.004). There was no significant difference in 25-hydroxy vitamin D levels comparing AN-BP and ARFID (*p* = 0.64). Additionally, when combining AN-R and AN-BP into one category, individuals with both subtypes of AN had a significantly higher 25-hydroxy vitamin D level compared to those with those with ARFID (F[1,294] = 4.53, *p* = 0.03, *η*^2^ = 0.015). Approximately 1/3 of individuals with ARFID (35%) were either deficient or insufficient in vitamin D, and 29% of individuals with AN were deficient or insufficient (*x*^*2*^[1] = 0.93, p = 0.34, ϕc = 0.05).

### Relationship between Nadir phosphorus and other variables of interest

Nadir phosphorus level showed a significant positive association with weight, BMI, %IBW, serum potassium, calcium, and prealbumin on admission (Table 3). A negative association was found with length of stay (*r*^*2*^ = − 0.170, *p* = 0.003), total number of tube-fed days (*r*^*2*^ = − 0.170, *p* = 0.003), and hemoglobin on admission (*r*^*2*^ = − 0.140, *p* = 0.014). A non-significant trend was found between use of tube feeding (dichotomized as present versus absent) and nadir phosphorus level (F[1,305] = 3.85, *p* = 0.051, *η*^2^ = 0.012). Nadir phosphorus level was not associated with age, weight gain per week during hospitalization, kcal prescribed on admission, duration of illness, or remaining laboratory studies (admission magnesium, AST, ALT, point-of-care blood glucose).

**Table 3:** Correlation between variables of interest and nadir serum phosphorus during hospitalization

	Whole sample (*n* = 307)		ARFID (*n* = 58)		AN (*n* = 249)	
	*r*	*p*	*r*	*p*	*r*	*p*
Admit weight (kg)	.201**	< .001	.199	.134	.203*	.001
Admit BMI (kg/m^2^)	.191*	.001	.249	.060	.180*	.004
Admit %IBW	.174*	.002	.279*	.034	.151*	.017
Weight gain (kg/week)	− .078	.175	− .020	.884	− .065	.306
Admit magnesium	− .109	.057	− .235	.076	− .093	.146
Admit hemoglobin	− .140*	.014	− .086	.522	− .083	.194
Admit prealbumin	.124	.031	.111	.408	.121	.059
Admit calcium	.216**	< .001	.105	.431	.193*	.002
Blood glucose POCT	− .014	.810	− .137	.311	− .013	.840

	*r*_*s*_	*p*	*r*_*s*_	*p*	*r*_*s*_	*p*
Age	− .077	.177	− .077	.568	− .079	.212
Length of stay	− .170*	.003	− .184	.166	− .161*	.011
Duration of illness (years)	− .027	.674	.199	.237	− .053	.448
Kcal on admission	.012	.838	− .078	.563	.050	.434
Admission vitamin D	.041	.477	.099	.464	.021	.748
Admission potassium	.247**	< .001	.338*	.010	.232**	< .001
AST	− .002	.972	.083	.544	− .029	.651
ALT	.000	.996	*.094*	.494	− .030	.638
Total number of tube fed days	− .170*	.003	− .116	.386	− .180*	.004

*ARFID* Avoidant/restrictive food intake disorder, *AN* anorexia nervosa, BMI Body mass index, *IBW* Ideal body weight, *POCT* Point of care test, *Kcal* Kilocalories, *AST* Aspartate transferase, *ALT* Alanine aminotransferase

^*^p ≤ .05; ** *p* < .001

### Relationship between Nadir phosphorus and vitamin D

For individuals with both AN and ARFID, there was no correlation between 25-hydroxy vitamin D level on admission and nadir serum phosphorus level (*p* = 0.447). There was a non-significant trend for individuals who were vitamin D deficient (≤ 20 ng/mL) to be more likely to become hypophosphatemic during admission than those whose 25-hydroxy vitamin D levels were greater than 20 ng/mL (either insufficient or sufficient; *x*^2^[1] = 3.796, *p* = 0.051, ϕc = 0.112). There was no difference in RH likelihood comparing across three categories of vitamin D (deficient, insufficient, and sufficient; *p* = 0.066, ϕc = 0.133) or when comparing individuals whose vitamin D was sufficient to those with either deficient or insufficient vitamin D levels (i.e. when vitamin D level was dichotomized as less than or greater than 30 ng/mL; *p* = 0.75, ϕc = 0.018). There were no differences observed between those with AN compared to those with ARFID (*p* = 0.066, ϕc = 0.158).

### Likelihood of refeeding hypophosphatemia

When analyzing phosphorus as a dichotomous variable (whether or not a threshold level of < 2.9 mg/dL was met), associations with variables of interest were the same as with nadir phosphorus level. A logistic regression model was created evaluating likelihood of hypophosphatemia included admission weight, length of stay, potassium, calcium, hemoglobin, prealbumin, total number of tube-fed days, 25-hydroxy vitamin D level and controlled for total dose of vitamin D supplement during hospitalization. The final model was significant (*x*^2^ (9) = 48.51, *p* < 0.001), and admission weight, potassium, hemoglobin, prealbumin, and calcium contributed significantly to the model. The model explained 20% (Nagelkerke R^2^) of the variance in refeeding hypophosphatemia and correctly identified 72% of the cases of RH. Odds ratios are found in Table 4.

**Table 4:** Odds of hypophosphatemia by associated risk factors (*N* = 301)

	Β	Exp (B)	Wald	*p*	95% confidence interval
Weight on admission (kg)	− .044	.957	4.74	.029*	.924–.994
Hemoglobin	.166	1.18	3.91	.049*	1.002–1.393
Calcium	− .814	.443	9.23	.002*	.262–.750
Prealbumin	− .046	.955	4.34	.037*	.914–.997
Potassium	− 1.065	.345	16.71	< .001**	.207–.575
Tube Feeding	.030	1.03	3.00	.083	.997–1.064
Vitamin D	.005	1.005	.372	.542	.9856–1.016
Length of stay	− .002	.903	.015	.998	.972–1.032

^*^p ≤ .05

^**^p < .001

### Moderation by vitamin D

**Fig. 1:**
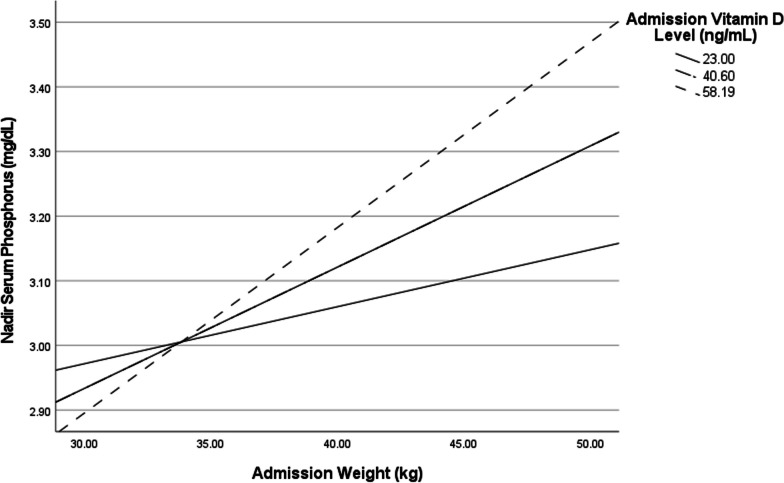
Moderation of the effect of vitamin D level on admission in the relationship between admission weight and phosphorus nadir

Vitamin D did not moderate the relationship between low body weight and nadir phosphorus level (*B* = 0.0006, SE = 0.0003, *p* = 0.056). However, the conditional effects show that this interaction was significant at higher levels of 25-hydroxy vitamin D, such that higher levels of vitamin D were associated with significantly higher phosphorus nadir level as weight increased (Fig. 1). This interaction was significant for 25-hydroxy vitamin D levels at the mean (40.6 ng/mL and higher but not for levels one standard deviation below the mean (23.0 ng/mL). For those whose vitamin D level was at least 40.6, nadir serum phosphorus was significantly higher than those whose vitamin D level was lower as admission weight increased (*p* = 0.0001). For individuals with low levels of vitamin D, there were no significant differences between nadir phosphorus level as admission weight increased (*p* = 0.17). No interaction effects were found between vitamin D and age, BMI, %IBW, duration of illness, potassium, magnesium, hemoglobin, prealbumin, calcium, or caloric prescription on the outcome of nadir phosphorus level.

## Discussion

Refeeding hypophosphatemia, a common complication during nutrition restoration for restrictive eating disorders [40], is a target of clinical monitoring and need for supplementation [41]. Despite awareness, close clinical monitoring, prophylaxis, and intervention, RH risk remains a poorly understood phenomenon. Consequently, there exists wide variability in the interventions utilized during nutrition rehabilitation [9, 42]. Additionally, clinicians may alter care while monitoring serum phosphorus, such as halting caloric intake increases, changing laboratory draw frequency, or increasing prophylactic phosphorus doses in response to down-trending serum phosphorus regardless of reaching RH threshold. Therefore, we examined both serum phosphorus nadir as well as whether or not the individual experienced hypophosphatemia for each study aim. This study is the first, to the authors’ knowledge, to examine the risk of RH in relation to 25-hydroxy vitamin D levels upon admission for a medical stabilization program among a cohort of severely ill individuals with AN and ARFID. Additionally, information about prevalence rates of vitamin D deficiency in AN and ARFID is limited. The results presented here describe both the prevalence of vitamin D deficiency, as well as risk of RH in the context of that finding.

Overall, there has been a paucity of research focusing on patients with ARFID. In this cohort, individuals with ARFID had significantly lower 25-hydroxy vitamin D levels than individuals with AN-R, and similar rates of deficiency and insufficiency in Vitamin D compared to individuals with AN-BP. These findings occurred despite no significant differences in patient characteristics including weight and BMI on admission. This result highlights the need for careful consideration of the nutritional state of individuals with ARFID, especially those who are demonstrating signs of severe malnutrition. Although ARFID notably includes a heterogeneous cluster of symptom presentation and nutritional intake profiles, previous studies have found that macro- and micronutrient intake in ARFID is significantly reduced compared to controls [43, 44]. However, the authors of those studies found no difference in vitamin D intake and did not specifically detail phosphorus intake. It remains unclear why individuals with ARFID had significantly lower 25-hydroxy vitamin D levels than individuals with AN-R in this cohort. Specific dietary sources of vitamin D such as eggs, yogurt, cheese, and fish may be consumed less frequently by individuals with sensory challenges and contribute to this phenomenon. However, generally accepted foods such as cereal fortified with vitamin D may offset the lack of intake from other sources. Additionally, it has been proposed that there is a high prevalence of ARFID in children with Autism Spectrum Disorders [45], which commonly present with severe food selectivity and associated vitamin D deficiency [46]. Future studies examining causes for low vitamin D in individuals with ARFID and AN should be explored, including environmental exposure. Research to date suggests that clinicians in both ambulatory and inpatient settings should consider individuals with ARFID as nutritionally fragile regardless of weight, and ARFID may warrant an approach similar to the thoughtful nutrition restoration that occurs in AN.

Low weight on admission has been identified as a risk factor for RH, yet low percent of variance in nadir serum phosphorus has been explained by low weight on admission [15]. Although recent position statements note that clinicians should have a high index of suspicion for RH in individuals who present at a low weight, have lost a large percent of body weight, or lost weight quickly [47], it remains unclear what additional factors intersect with low weight to create greatest risk. In this work, 25-hydroxy vitamin D at higher levels (40.6 ng/mL or greater) significantly moderated the relationship between admission weight and nadir serum phosphorus level. This suggests a possibility that vitamin D may be protective against the development of RH, although significant effects at higher weights may be of less clinical utility. Further research is necessary to elucidate the relationship between weight and vitamin D as it pertains to RH.

The findings of this study point toward a need to further investigate a possible link between vitamin D and serum phosphorus levels as components of a negative feedback loop that maintains phosphorus and calcium homeostasis. In this system, serum phosphorus levels are maintained through gut absorption mediated by vitamin D, parathyroid hormone stimulation of renal phosphorus and calcitriol excretion, as well as parathyroid hormone influence on fibroblast growth factor-23-mediated bone absorption of calcium and phosphorus [49]. In this study, we found a non-significant trend toward a higher likelihood of phosphorus below 2.9 mg/dL in those with vitamin D deficiency. Additionally, there was evidence of moderation between weight on admission and serum phosphorus nadir for individuals with higher levels of 25-hydroxy vitamin D. Finally, low levels of serum calcium on admission were significantly associated with lower nadir phosphorus levels. Together, this suggests that further research is needed to clarify the negative feedback loop that promotes calcium and phosphorus homeostasis in the context of RH in eating disorders. Future work should focus on further understanding exactly what level of 25-hydroxy vitamin D, if any, marks a protective benchmark against the development of RH.

This work is not without limitations. First, the study population was unique in its severity and may not represent mild-moderate illness. The sample was overwhelmingly female and therefore comparative analysis between sexes was not powered in this cohort. This sample did not represent transgender individuals who may require additional consideration in the context of hormonal treatment as seen in a recent case report of recurrent and persistent RH [50]. Additionally, 89% (*n* = 275) of the sample had a starting caloric prescription of 1400 kcal, with only 24 individuals receiving 1800 kcal on admission, 2 individuals receiving 2000 kcal on admission, and 6 receiving 2100–4000 kcal. Therefore, the non-significant relationship between admission kcal and RH should be interpreted with caution. Future prospective work should include higher caloric variability in a similarly medically fragile, very low-weighted cohort. Correlations presented here, generally, should be interpreted with caution as there were no corrections performed for multiple comparisons. The present sample was comprised of individuals admitted to a medical critical care unit with a mean average admission weight lower than what may be found in other institutions; however, individuals with substantial weight loss, as in atypical AN, exhibit similar physiologic outcomes to those with AN [48]. Research on vitamin D in individuals with atypical AN may be of importance in further classifying risk of RH. Additionally, we did not include either day of recorded RH or weight suppression in the present study, which may be of interest with regards to RH outcomes. Finally, two key components of this negative feedback loop, parathyroid hormone and fibroblast growth factor-23, were not available in existing medical records and thus were not included in analyses. Further work is necessary to investigate the role of parathyroid hormone and fibroblast growth factor-23 in maintaining phosphorus levels during nutritional restoration.

## Conclusions

This study describes the prevalence of vitamin D deficiency and its associations with RH in severely malnourished inpatients with AN and ARFID. Over 35% of individuals in this cohort had hypophosphatemia over the course of admission with no differences between groups. Nadir phosphorus was associated with lower weight, BMI, % IBW, serum potassium, calcium, and prealbumin and higher hemoglobin on admission, longer length of stay, and greater number of days receiving tube feedings. Individuals with ARFID had significantly lower 25-hydroxy vitamin D levels on admission than individuals with AN, but were not more likely to be vitamin D deficient. Higher, but not lower, levels of 25-hydroxy vitamin D on admission significantly moderated the relationship between admission weight and serum phosphorus nadir.

This study highlights that a substantial proportion of individuals with AN and ARFID admitted for medical stabilization may be deficient or insufficient in vitamin D, and that individuals with ARFID may be as susceptible to both vitamin D deficiency and RH as those with AN. Screening for 25-hydroxy vitamin D on admission may be an important component of clinical care for malnourished individuals with AN and ARFID. Further work is needed to create clinical scoring systems to facilitate identification of risk of RH and prevent development of RH and refeeding syndrome.

## Data Availability

The data that support the findings of this study are available from the corresponding author upon request.

## Abbreviations

AN: Anorexia nervosa
ANOVA: Analysis of variance
ARFID: Avoidant/restrictive food intake disorder
ALT: Alanine transaminase
AST: Aspartate aminotransferase
BMI: Body mass index
%IBW: Percent ideal body weight
RH: Refeeding hypophosphatemia
